# A Successful Mother and Neonate Outcome for a Woman with Essential Thrombocytosis and FV Leiden Heterozygosity

**DOI:** 10.1155/2016/7041686

**Published:** 2016-03-31

**Authors:** Marianna Politou, Serena Valsami, Irontianta Gkorezi-Ntavela, Vasilios Telonis, Efrosyni Merkouri, Panagiotis Christopoulos

**Affiliations:** ^1^Blood Transfusion Department, Aretaieion Hospital, National and Kapodistrian Athens University Medical School, 11528 Athens, Greece; ^2^Coagulation Laboratory, Iatriko Kentro Hospital, 11525 Athens, Greece; ^3^2nd Department of Obstetrics and Gynecology, Aretaieion Hospital, National and Kapodistrian Athens University Medical School, 11528 Athens, Greece

## Abstract

Essential thrombocytosis (ET) and FV Leiden heterozygosity represent an acquired and hereditable hypercoagulable state, respectively. An uncommon case of coexistence of ET and FV Leiden heterozygosity in a 36-year-old pregnant woman and her successful pregnancy outcome is described. She was considered to be at high risk of thrombosis during her pregnancy and she was treated with both prophylactic dose of LMWH and aspirin daily throughout her pregnancy and for a 6-week period postpartum. The efficacy of the anticoagulation treatment was monitored in various time points not only by measuring anti-Xa levels and D-Dimers but also with new coagulation methods such as rotation thromboelastometry and multiplate. Global assessment of coagulation using additional newer laboratory tests might prove useful in monitoring coagulation pregnancies at high risk for thrombosis.

## 1. Introduction

Essential thrombocytosis (ET) is a myeloproliferative neoplasm characterized by thrombocytosis which is at high risk for thrombotic events. Two-thirds of the patients harbor the JAK2V617F mutation [1]. Conversely, FV Leiden is a common and mild form of hereditary thrombophilia [2, 3]. We here report a case of a successful pregnancy outcome for a woman with ET and FV Leiden heterozygosity.

## 2. Presentation of Case

A 36-year-old primigravida was referred from her gynecologist to the hematologist, at 6 weeks of gestation, for consultation regarding anticoagulation during pregnancy. The patient was diagnosed with ET 5 years priorly and was positive for JAK2V617F mutation. She was previously treated with Anagrelide but was currently on low-dose aspirin (80 mg/d). The year preceding pregnancy her PLT count ranged from 500 to 650 K/*μ*L. She was also found to be heterozygous for FV Leiden (her father had unprovoked DVT). She did not have other risk factors for thrombosis. Her body weight was 62 Kg. The rest of her medical history was unremarkable.

The patient was started on prophylactic dose of LMWH (4500 IU tinzaparin) along with aspirin 80 mgr daily. The platelet count during pregnancy never exceeded 600 K/*μ*L with a range from 480 to 570 K/*μ*L.

D-Dimer and anti-Xa levels were measured at 16th, 22nd, 28th, and 32nd weeks of gestation. Anti-Xa levels were within normal range for prophylactic LMWH dose (0.1–0.4 IU/mL), while values for D-Dimers were 730, 910, 1200, and 1480 *μ*g/mL, respectively.

**Figure 1:**
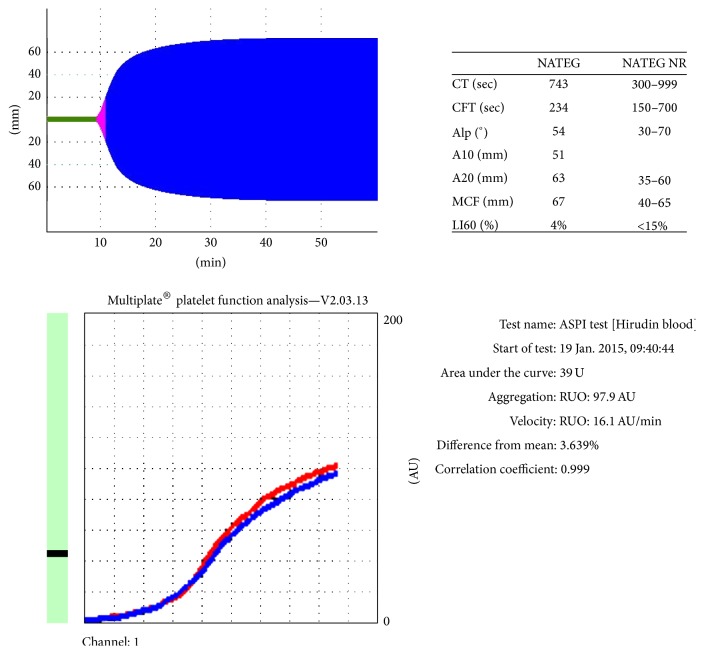
Rotational thromboelastometry: NATEM and multiplate ASPI performed on 22 weeks of gestation. ROTEM: clotting time (CT) for ROTEM is defined as the time in minutes it takes for the trace to reach an amplitude of 2 mm. Clot formation time (CFT) is defined as the time necessary for clot amplitude to increase from 2 to 20 mm. Angle (*α*) is determined by creating a tangent line from the point of clot initiation (CT) to the slope of the developing curve. Maximum clot firmness (MCF) is the peak amplitude (strength) of the clot. Lysis Index 30 (LI30) is the percent reduction in MCF that exists when amplitude is measured 30 min after CT is detected. Multiplate: results are expressed in AUC (area under the curve = area under the aggregation curve) units (U) and are recorded against time. The AUC reflects overall platelet activity, which is affected by the height of the aggregation curve. Agonists tested multiplate ASPI (arachidonic acid; final concentration, 0.5 mmol/L).

A rotational thromboelastometry (ROTEM) (ROTEM®; Munich, Germany) was performed before the start of LMWH and during the 22nd week of gestation. A NATEM (nonactivated TEM) test was performed with ROTEM and the parameters CT (closure time), CFT, an angle, and MCF (maximum clot firmness) were evaluated (Figure 1). During the 6th week of gestation, CT was 421 sec, CFT was 203 sec, and MCF was 67, while during the 22nd week of gestation, the values were 743 sec, 234 sec, and 67, respectively, and a multiplate test was also performed. Platelet aggregation was assessed on the Multiplate Analyzer (Roche Diagnostics, Mannheim, Germany) using arachidonic acid (multiplate ASPI: 0.5 mM). The inhibition is expressed as AUC (area under the curve) units (Figure 1).

The pregnancy was uneventful and ultrasound assessments were normal. At 37 + 4 weeks an elective Cesarean Section was performed due to premature rapture of membranes, and a healthy female neonate with birth weight of 3300 gr was born, with proper management of peripartum anticoagulation. Both the mother and the neonate were discharged on day 7 in good health. The mother received anticoagulation treatment with both LMWH and aspirin at the same dose (tinzaparin 4500 IU plus aspirin 80 mg daily) for a 6-week period postpartum.

## 3. Discussion

ET is associated with pregnancy complications. The life birth rate in women with ET ranges from 60 to 70% and first trimester abortions occur in one-third of pregnancies. Late pregnancy complications such as preeclampsia, placental abruption, intrauterine death or stillbirth, and IUGR are less frequent [4].

Pregnant women with ET can be characterized as low risk or high risk on the basis of history of thrombosis, bleeding, or complications in previous pregnancies [5]. Our patient had no history of thrombosis/bleeding and she was a primigravida. Thus, in terms of ET, she could be characterized as a low risk pregnancy and she could be managed only with low-dose aspirin and LMWH postpartum. Our patient was JAK2V617F positive but the role of the mutation for predicting pregnancy complications in ET women is still questionable.

Although FV Leiden is associated with increased risk for pregnancy complications, heterozygous FV Leiden mutation does not justify the administration of anticoagulation therapy in order to avoid pregnancy complications. A heterozygous FV Leiden pregnant woman with a positive family history for thrombosis is associated with a low risk of thrombosis that could only justify the use of LMWH prophylactically postpartum [6]. Due to the simultaneous presence of FV Leiden and ET the patient was considered as high risk and was treated accordingly with a combination of aspirin and LMWH during pregnancy and 6 weeks after delivery. Cytoreduction with INF-*α* was also kept as a therapeutic option in case of marked thrombocytosis [2, 6]. The platelet count in our patient never exceeded 500 K/*μ*L which is in support of the spontaneous reduction of platelet count classically observed in pregnant women with ET.

There are no specific guidelines for either the monitoring of a pregnant woman on double anticoagulation therapy or the modification of the dose of the anticoagulants. The choice of the optimum dose has to ensure that the woman is adequately anticoagulated without being exposed to an increased risk of bleeding. Anti-Xa levels monitoring during pregnancy is supported when complicated by renal failure or extreme weight gain [6].

D-Dimers are a good marker of coagulation and fibrinolytic system activation. D-Dimers normally increase with gestational age and nomograms adapted for each trimester of pregnancy should be consulted in order to evaluate D-Dimers results [7].

ROTEM is a point of care (POC) viscoelastic test of haemostasis in whole blood: it assesses clot formation and dissolution kinetics and strength in real time. ROTEM is a global coagulation test since clot formation and resolution are the result of the interactions of coagulation factors, red blood cells, platelets, and anticoagulants. It can quickly provide information for a patient's thrombotic or bleeding tendency. In obstetrics, thromboelastography has been successfully used for the management of postpartum haemorrhage (PPH) where the usefulness of standard laboratory tests is limited. ROTEM based algorithms for diagnosis and treatment of specific coagulation deficiencies in patients with PPH have also been suggested. Moreover, it is very helpful in determining suitability for neuraxial anaesthesia [8].

ROTEM has also been used to monitor hypercoagulability during pregnancy, but since hypercoagulability seems to increase with gestational age, ROTEM results should be interpreted with caution in pregnancy. ROTEM range values in each trimester of pregnancy are different compared to nonpregnant women, but these values are not formally established [9, 10]. In our case we decided to use ROTEM in order to assess simultaneously the effect of both drugs with one test and determine whether the patient is hypercoagulable or haemorrhagic. ROTEM parameters in our patient were within normal range, only MCF was slightly higher, 67 mm (normal range 40–65 mm). The patient was in 22nd week and thus she was slightly hypercoagulable.

Multiplate Analyzer rapidly evaluates platelet function. Multiplate ASPI test evaluates platelet aggregation as a response to aspirin. With the known variability in individual responsiveness to aspirin, multiplate can be useful at identifying patients with resistance to aspirin [11]. The patient was on aspirin when she became pregnant and a multiplate ASPI performed the year preceding pregnancy was multiplate ASPI < 40. At 22nd week of gestation multiplate ASPI was 39 U and thus the aspirin dose was kept the same throughout pregnancy.

Coexistence of ET and FV Leiden is an uncommon thrombotic predisposition. Pregnant women with both entities should be regarded as high risk for thrombosis and treatment with both aspirin and LMWH seems to be a reasonable option. Monitoring the patient with D-Dimers, thromboelastometry, and multiplate can help the clinician to adjust the right dose of anticoagulation [12].
